# Association of recurrent mutations in *BRCA1, BRCA2, RAD51C, PALB2*, and *CHEK2* with the risk of borderline ovarian tumor

**DOI:** 10.1186/s13053-022-00218-0

**Published:** 2022-03-21

**Authors:** Alicja Ogrodniczak, Janusz Menkiszak, Jacek Gronwald, Joanna Tomiczek-Szwiec, Marek Szwiec, Cezary Cybulski, Tadeusz Dębniak, Tomasz Huzarski, Aleksandra Tołoczko-Grabarek, Tomasz Byrski, Katarzyna Białkowska, Karolina Prajzendanc, Piotr Baszuk, Jan Lubiński, Anna Jakubowska

**Affiliations:** 1 Department of Genetics and Pathology, Pomeranian Medical University in Szczecin, ul. Unii Lubelskiej 1, 71-252 Szczecin, Poland; 2 Department of Gynecological Surgery and Gynecological Oncology of Adults and Adolescents, Pomeranian Medical University in Szczecin, Szczecin, Poland; 3 Department of Histology, Department of Biology and Genetics, Faculty of Medicine, University of Opole, Opole, Poland; 4 Department of Surgery and Oncology, University of Zielona Góra, Zielona Góra, Poland; 5 Department of Clinical Genetics and Pathology, University of Zielona Góra, Zielona Góra, Poland; 6 Department of Oncology and Chemotherapy, Pomeranian Medical University in Szczecin, Szczecin, Poland; 7 Independent Laboratory of Molecular Biology and Genetic Diagnostics, Pomeranian Medical University in Szczecin, Szczecin, Poland

**Keywords:** Borderline ovarian tumor, Low-grade ovarian cancer, Recurrent mutations, *BRCA1*, *BRCA2*, *RAD51C*, *PALB2*, *CHEK2*

## Abstract

**Background:**

There are several genes associated with ovarian cancer risk. Molecular changes in borderline ovarian tumor (BOT) indicate linkage of this disease to type I ovarian tumors (low-grade ovarian carcinomas). This study determined the prevalence and association of mutations in *BRCA1, BRCA2, PALB2, RAD51C*, and *CHEK2* with the risk of BOTs.

**Methods:**

The study group consisted of 102 patients with histologically confirmed BOT and 1743 healthy controls. In addition, 167 cases with ovarian cancer G1 were analyzed. The analyses included genotyping of 21 founder and recurrent mutations localized in 5 genes (*BRCA1, BRCA2, PALB2, RAD51C,* and *CHEK2).* The risk for developing BOT and low-grade ovarian cancer, as well as the association of tested mutations with survival, was estimated.

**Results:**

The *CHEK2* missense mutation (c.470T>C) was associated with 2-times increased risk of BOT (OR=2.05, *p*=0.03), at an earlier age at diagnosis and about 10% worse rate of a 10-year survival. Mutations in *BRCA1* and *PALB2* were associated with a high risk of ovarian cancer G1 (OR=8.53, *p*=0.005 and OR=7.03, *p*=0.03, respectively) and were related to worse all-cause survival for *BRCA1* carriers (HR=4.73, 95%CI 1.45–15.43, *p*=0.01).

**Conclusions:**

Results suggest that *CHEK2 *(c.470T>C) may possibly play a role in the pathogenesis of BOT, but due to the low number of BOT patients, obtained results should be considered as preliminary. Larger more in-depth studies are required.

## Background

Borderline ovarian tumors (BOTs) are neoplasms of epithelial origin, characterized by upregulated cellular proliferation and the presence of nuclear abnormalities, but in contrast to ovarian cancer, they do not show infiltrative growth patterns [1, 2]. Borderline tumors constitute approximately 10–20% of all epithelial ovarian neoplasms [3] and are histologically classified based on the epithelial cell type, similarly to invasive carcinomas. The most common subtypes are serous (50%) and mucinous (45%), while the less common include endometrioid, clear cell, seromucinous, and borderline Brenner tumor [1–3]. BOTs are usually limited to the ovaries and are diagnosed at FIGO stage I [4]. Although the prognosis in BOT patients is generally excellent, serous borderline tumors can implant on peritoneal surfaces and progress to low-grade serous ovarian carcinoma [2, 3]. There are several genes associated with the risk of ovarian cancer [5–9]; however, very little is known about germline mutations related to BOTs [10–17].

Most cancer predisposition genes are considered molecularly heterogeneous, displaying hundreds of different disease-causing sequence alterations. However, in certain populations, founder mutations can be identified, which are located within a genomic region that is in linkage disequilibrium and therefore segregates as a unit. the founder mutations are inherited and often remain restricted to one or a few populations or specific geographic regions. The Polish population has been recognized as relatively homogeneous in terms of the genetic make-up. Studies on genetic predisposition to cancer diseases revealed the existence of a number of founder alleles and recurrent mutations in several genes, including *BRCA1, CHEK2*, and *PALB2* genes [18–22]. Recently, 21 founder and recurrent mutations in *BRCA1/2, PALB2, RAD51C*, and *CHEK2* genes have been analyzed in a large Polish case-control study including 2270 ovarian cancer patients and 1743 controls. Results have shown a significant association of mutations in *BRCA1/2* and *RAD51C* with cancer risk [5].

In the current study, we analyzed the prevalence of 21 recurrent germline mutations in *BRCA1, BRCA2, RAD51C, PALB2*, and *CHEK2* genes among Polish patients with BOTs and decided to determine whether are associated with the risk of developing this tumor.

## Methods

### Study group

The study group consisted of 102 patients with histologically confirmed BOT diagnosed between 1975 and 2018 in Independent Public Clinical Hospital No. 2 of the Pomeranian Medical University in Szczecin and were recruited between 2001 and 2017 years. The mean age of BOT patients at the time of recruitment was 49 years (range 17–83 years). Based on reported pathological and molecular similarities of BOTs and low-grade invasive ovarian tumors [23, 24], 167 women with low-grade ovarian cancer (grade 1—G1) were additionally included in analyses. The patients with ovarian cancer G1 were selected from a group of 2270 consecutive ovarian cancer cases described earlier [5]. The obtained clinical information included age at diagnosis, tumor histology, and family history of ovarian cancers. Data on the vital status was collected from the Ministry of Digital Affairs in July 2019. The characteristics of patients are presented in Table 1.

**Table 1:** Characteristics of patients in study groups

Feature	*BOT cases, ***n***=102 (%)	Ovarian cancer G1 cases, ***n***=167 (%)
Mean age at diagnosis (range)	47.76 (17–83)	54.25 (19–84)
Mean follow up in months (range)	126 (10–528)	82 (1–216)
The histological type of tumor		
Serous	61 (59.8%)	79 (47.3%)
Mucinous	22 (21.6%)	38 (22.8%)
Endometrioid	0 (0%)	36 (21.6%)
Clear-cell	0 (0%)	2 (1.2%)
Other or undefined/mixed	1 (0.1%)	2 (1.2%)
Missing	18 (17.6%)	10 (6.0%)
Family history of OC		
Yes	12 (11.8%)	8 (4.8%)
No	88 (86.3%)	152 (91.0%)
Missing	2 (2.0%)	7 (4.2%)
Death		
Yes	10 (9.8%)	44 (26.3%)
No	88 (86.3%)	113 (67.7%)
Missing	4 (3.9%)	10 (6.0%)

*BOT borderline ovarian tumor

The control group consisted of 1743 healthy women selected from the registry of the International Hereditary Cancer Center in Szczecin, as described earlier [5]. Briefly, they were women recruited in the West Pomerania region between 2002 and 2019 year. For all controls, demographic and clinical data, as well as information about family history of ovarian cancer, were collected. The mean age of controls at the time of recruitment was 55.8 years (range 18–90 years). Healthy controls were followed since the time of recruitment, and the mean follow-up was 26.1 months (0–213 months).

### Sample preparation molecular analysis

A blood sample was obtained from all cases and controls, and the DNA was isolated using a previously described method [25]. DNA samples were stored at 4 °C prior to genotyping. The analysis encompassed genotyping of 21 recurrent/founder germline mutations localized in five genes: *BRCA1* (9 mutations)*, BRCA2* (4 mutations)*, RAD51C* (3 mutations)*, PALB2* (2 mutations), and *CHEK2* (3 mutations), as listed in Table 2.

**Table 2:** Recurrent mutations tested in *BRCA1, BRCA2, RAD51C, PALB2,* and *CHEK2* genes

Gene	cDNA	Protein change	Molecular consequence
***BRCA1***	*5*382insC (c.5266dupC)	p.Gln1756fs	Fr﻿ameshi﻿f﻿t
	300T>G (c.181T>G)	p.Cys61Gly	Missense
	4153delA (c.4035delA)	p.Glu1346fs	Frameshift
	1806C>T (c.1687C>T)	p.Gln563Ter	Nonsense
	185delAG (c.68_69delAG)	p.Glu23fs	Frameshift
	3819del5 (c.3700_3704del5)	p.Val1234fs	Frameshift
	3875del4 (c.3756_3759delGTCT)	p.Ser1253fs	Frameshift
	5370C>T (c.5251C>T)	p.Arg1751Ter	Nonsense
	794delT (c.794_795delCT)	p.Ser265Cysfs	Frameshift
***BRCA2***	4075delGT (c.3847_3848delGT)	p.Val1283fs	Frameshift
	8138del5 (c.7913_7917delTTCCT)	p.Ala2637_Phe2638insTer	Nonsense
	886delGT (c.658_659del)	p.Val220fs	Frameshift
	6174delT (c.5946delT)	p.Ser1982fs	Frameshift
***RAD51C***	c.905-2_905-1delAG	p.Glu303TrpfsX41	Skipping of exon 7
	c.577C>T	p.Arg193Ter	Nonsense
	c.502A>T	p.Arg168Ter	Nonsense
***PALB2***	c.172_175delTTGT	p.Gln60fs	Frameshift
	c.509_510delG>A	p.Arg170fs	Frameshift
***CHEK2*** PTT Missense	c.1100delC*	p.Thr367fs	Frameshift
	c.444+1G>A*	p.E149IfsX6	Skipping of exon 2
	c.470T>C	p.Ile157Thr	Missense

*PTT protein truncating mutations

Genotyping was performed as described earlier [5]. All mutations, with the exception of c.5266dupC in *BRCA1*, were genotyped by a real-time PCR using customized TaqMan Assays on LightCycler Real-Time PCR 480 System (Real-Time PCR System, Roche Diagnostics, Indianapolis, IN, USA), following the standard protocol. The reaction mix for analysis of each sample included GoTaq® Probe qPCR Master Mix (Promega, Madison, WI, USA), TaqMan Genotyping Assays × 40 or TaqMan Genotyping Assays × 80 (Applied Biosystems, Foster City, CA, USA), and deionized water (Promega, Madison, WI, USA). Samples were analyzed on 384-well plates. On each analyzed plate 3 control samples were included: positive and negative for tested mutations and a water-blind control. The genotyping was performed using LightCycler 480 Instrument, and data were analyzed using LightCycler 480 Basic Software Version 1.5 (Roche Diagnostics, Indianapolis, IN, USA). The analysis for c.5266dupC mutation in *BRCA1* was performed using allele-specific amplification PCR (ASA-PCR) as described previously [20]. The sequences of primers and probes used for genotyping are available on request.

All study participants provided written informed consent. The study was approved by the Ethics Committee of the Pomeranian Medical University in Szczecin (protocol code KB-0012/153/16 (BN-001/33/04), 20.12.2016).

### Statistical analysis

The results of genotyping 21 mutations localized in 5 genes were used to estimate the prevalence and an association of tested mutation with the risk of BOTs and low-grade ovarian cancer. The ORs and corresponding 95% confidence intervals (95% CIs) were calculated using a logistic regression model.

The information about specific causes of death was not available, and therefore, the all-cause survival was calculated for all patients which were followed from the date of diagnosis until death from any cause. The hazard ratios (HRs) were estimated for mutation carriers in comparison with non-carriers using Cox proportional hazards. In addition, the 5- and 10-year survival among the *BRCA1*, *BRCA2*, *RAD51C*, *PALB2*, and *CHEK2* mutation carriers was calculated.

All analyses were done separately for each gene, *BRCA1*, *BRCA2*, *RAD51C*, *PALB2*, and *CHEK2* (separately for two protein-truncating mutations and one missense mutation, as shown in Table 2) using R Project for Statistical Computing (R version: 4.0.3). The *p*-values less than 0.05 were recognized as statistically significant.

## Results

### Association of *BRCA1, BRCA2, RAD51C, PALB2*, and *CHEK2* mutations with BOT risk

Genotyping of 21 mutations in *BRCA1, BRCA2, RAD51C, PALB2,* and *CHEK2* genes was performed for 102 BOT patients, 167 cases of ovarian cancer G1, and 1743 healthy controls. The prevalence of mutations was similar in both tested groups of ovarian tumor patients: 12.75% in BOT (13 out of 102 cases) and 12.57% in ovarian cancer G1 cases (21 out of 167). In the control group, mutations were detected in 6.77% (118 out of 1743) individuals.

Mutations in *BRCA1* and *PALB2* genes were significantly associated with the high risk of ovarian cancer G1, while *CHEK2* missense mutation (c.470T>C) was associated with 2-times elevated risk of BOT. The distributions of mutations in *BRCA1, BRCA2, RAD51C, PALB2, CHEK2* genes, and the ORs associated with the risk of ovarian tumors are shown in Table 3.

**Table 3:** The prevalence and association of tested 21 founder/recurrent mutations in *BRCA1*, *BRCA2*, *RAD51C*, *PALB2*, and *CHEK2* genes with the risk of BOT and low-grade ovarian cancer

**Gene**	**Controls, *n=1743 (%)***	**Cases**	
		BOT, ***n***=102 (%)	Ovarian cancer G1, ***n***=167 (%)
***BRCA1***	5 (0.29)	0 (0.00)	4 (2.40)
OR (95% CI) *p* value	ref.	-	**8.53 (2.09–32.54)** **0.005**
***BRCA2***	0 (0.00)	1 (0.98)	1 (0.60)
OR (95% CI) *p* value	ref.	-	-
***RAD51C***	3 (0.17)	0 (0.00)	0 (0.00)
OR (95% CI) *p* value	ref.	-	-
***PALB2***	3 (0.17)	0 (0.00)	2 (1.20)
OR (95% CI) *p* value	ref.	-	**7.03 (0.92–42.72)** **0.03**
***CHEK2*** *PTT*	10 (0.57)	1 (0.98)	1 (0.60)
OR (95% CI) *p* value	ref.	1.72 (0.09**–**9.09) 0.61	1.04 (0.06**–**5.5) 0.97
missense mutation (c.470T>C) OR (95% CI) *p* value	97 (5.57) ref.	11 (10.78) **2.05 (1.01–3.81)** **0.03**	13 (7.78) 1.43 (0.75**–**2.53) 0.24

The mean age at diagnosis of BOT was lower among cases with any detected mutation than without mutation: 45 years vs. 49 years, respectively. Carriers of *CHEK2* missense mutation (c.470T>C) were diagnosed about 10 years younger than non-carriers (38 years vs. 49 years).

The mean age at diagnosis of ovarian cancer G1 was similar for carriers of any mutation and non-carriers: 55 years and 54 years, respectively.

Among *CHEK2* missense (c.470T>C) mutation carriers, the family history of ovarian cancer was present in 3 (27.3%) patients with BOT and none with ovarian cancer G1.

There was no association of tested mutations with the histological type of ovarian tumor, neither in BOT nor ovarian cancer G1 patients.

### All-cause survival

Information on all-cause survival was available from 98 out of 102 BOT cases, and among them, 10 deaths (10.2%) were recorded. There was no association of all-cause survival with mutations in any of the 5 tested genes. The 5-year survival rate for all BOT patients was 90.48% and was similar for any mutation carriers and non-carriers (90% and 89.93%, respectively); the 10-year survival rate was 88.89% and was about 10% lower for mutation carriers (80%) than for non-carriers (89.80%).

In a group of ovarian cancer G1 patients, the information about all-cause survival was available from 157 of 167 cases, and 44 deaths (28%) were recorded. The estimated HRs for all-cause survival among mutation carriers were not statistically significant for any of the tested genes except *BRCA1* (HR = 4.73, 95%CI 1.45–15.43, *p* = 0.01). The 5-year survival rate for all patients was 71.32%, and the 10-year survival rate was 59.36%. There was no difference between mutation carriers and non-carriers (5 years: 69.23% and 69.72%; 10 years: 60% and 58.62%).

## Discussion

Borderline ovarian tumors (BOTs) represent a heterogeneous group of noninvasive tumors of uncertain malignant potential. They have characteristic histology and usually occur in younger women, before 40–45 years of age. The majority of BOT is diagnosed at the early stage and has a favorable prognosis [1–3]. However, the symptomatic recurrence rate varies between 3% [26] and 34% [27] and is associated with decreased survival [28]. The molecular changes in BOTs indicate linkage of this disease to type I ovarian tumors (low-grade ovarian carcinomas) [2, 3, 29, 30]. Recently, we found that pathogenic founder/recurrent germline mutations in 4 genes (*BRCA1, BRCA2, RAD51C, PALB2*) are responsible for 12.5% of ovarian cancer cases among unselected patients in the Polish population [5]. In the present study, we aimed to estimate the prevalence of 21 founder/recurrent germline mutations in 5 genes (*BRCA1, BRCA2, RAD51C, PALB2,* and *CHEK2*) among Polish patients with BOTs and to assess an association of these mutations with the risk of development of BOTs. Because of morphological similarities of borderline tumors and low-grade ovarian cancer [2, 3], we also included in the analysis 167 patients with ovarian cancer G1. Pathogenic mutations were detected in 12.74% of BOT (13 out of 102 cases), in 12.57% of ovarian cancer cases (21 out of 167 cases), and in 6.71% controls (117 out of 1743 women).

In the group of BOT patients, the most common was the missense mutation (c.470T>C) in the CHEK2 gene. The frequency of this mutation was higher in BOTs than in controls (10.8% vs. 5.6%), which corresponded to a statistically significant 2-times elevated risk of BOT (OR = 2.05, *p* = 0.03)*.* The prevalence of other tested recurrent mutations in *BRCA1, BRCA2, RAD51C,* and *PALB2* and two protein-truncating mutations in *CHEK2* among BOT patients was very low and was insufficient to perform a reliable association analysis (Table 3).

In a group of ovarian cancer G1 patients, statistically significant association with cancer risk was detected for mutations in *BRCA1* (OR = 8.53, *p* = 0.005) and *PALB2* (OR = 7.03, *p* = 0.03) genes; however, it should be noted that the number of mutation carriers was very limited (4 cases with *BRCA1* and 2 cases with *PALB2*). The *CHEK2 *mutations, including missense and two protein-truncating, were not significantly associated with cancer risk (Table 3).

The *CHEK2 *missense mutation (c.470T>C) was previously analyzed in another study of 539 ovarian cystadenomas, 122borderline ovarian tumors, and 447 ovarian cancer cases, including 88 low-grade (G1) tumors [15]*. *In that study, the significant association of *CHEK2 *missense mutation (c.470T>C) with the risk of non-invasive tumors (OR = 1.7, *p* = 0.005 for ovarian cystadenoma and OR= 2.6, *p* = 0.002 for BOTs) and borderline significant correlation with low-grade ovarian cancer (OR=2.1, *p* = 0.04) were reported. In our study, the association of *CHEK2 *missense mutation (c.470T>C) was statistically significant for BOT risk, but not for the risk of ovarian cancer G1 (Table 3). The possible explanation of the discrepancy in the above results may be the difference in a number of tested low-grade ovarian cancer cases, which was almost 2-times larger in our study (167 cases) than in previous analysis (88 cases)*.* The mutations in the *CHEK2* gene were already reported to correlate with the risk of prostate and breast cancer [20, 21, 31], but not with ovarian cancer [5, 21, 32]. The results of this study seem to support this observation and may extend it, suggesting a possible association of *CHEK2 *missense (c.470T>C) mutation with the 2-times increased risk of BOT*.*

Mutations in *BRCA1, BRCA2, RAD51C,* and *PALB2* genes were already shown to correlate with the risk of ovarian cancer [5–9], but not with BOTs [10–14, 33]. The two studies conducted on the Jewish population (including 117 BOT and 161 ovarian cancer cases, as well as 46 BOT and 59 ovarian cancer cases, respectively) showed a much lower incidence of thfoundersnder *BRCA1/2* mutations in BOT patients than in invasive early-stage ovarian carcinoma patients—the prevalence varied between 2.2 and 4.3% in BOT cases and 24.2–32% in ovarian cancer cases [10, 11]. In other studies, from Norway (190 BOT and 478 ovarian cancer patients) and Canada (134 BOT and 515 ovarian cancer patients), the *BRCA1/2* mutations were detected in 4% and 11.7% invasive cases, respectively, but none of the patients with BOT [12, 13]. There were performed several other smaller studies, in which only single cases of *BRCA1/2* mutations among BOT patients were detected, and the cumulative prevalence in all tested patients was 1.3% for *BRCA1* and 0.2% for *BRCA2* genes [33]. In contrary to the reports mentioned above, in a study of 1333 Czech ovarian cancer patients and 152 borderline ovarian tumor cases recruited from seven centers, the prevalence of *BRCA1/2* mutations was similar in high-grade ovarian cancers and BOT cases (30.9% and 28.9%) [14]. There is no obvious explanation for such a high frequency of *BRCA1/2* mutations among patients with BOT. It may be the consequence of several factors, such as differences in sample size or population-related differences. It should be also considered that pathological differentiation of BOTs from invasive tumors is not easy and may differ, especially when evaluation is done by pathologists from different centers [34, 35]. In our study, the 102 patients with histologically confirmed BOTs were diagnosed in one hospital, and the histology was evaluated and reviewed by 3 independent pathologists, which indicates a high-quality assurance of pathology diagnosis.

In our analysis, the mean age at diagnosis was lower among BOT patients than among low-grade ovarian cancer patients (47.76 vs. 54.25; Table 1), which is consistent with reported data [3, 36, 37]. The occurrence of any detected mutation in *BRCA1, BRCA2, RAD51C, PALB2*, and *CHEK2* genes was in general associated with earlier age of diagnosis of BOT, when compared to non-carriers (45 years vs. 49 years) and was 10 years younger in carriers of *CHEK2* missense mutation (c.470T>C) than in BOT patients without mutation (38 years vs. 49 years). This observation is consistent with a previous study which also reported a correlation of *CHEK2 *missense (c.470T>C) mutation with earlier age of diagnosis among BOT patients [15].

The all-cause survival, as it was expected, was better among BOT patients than ovarian cancer cases (5-year survival rate: 90.48 vs. 71.32% and 10-year survival rate: 88.89 vs. 59.36%), which is consistent with other studies reporting favorable prognosis for patients with borderline ovarian tumors [4, 28, 38–40]. There was no significant association of mutation status among BOT patients and death. However, the 10-year survival rate was lower for mutation carriers (80%) than for non-carriers (89.8%). Among patients with ovarian cancer G1, the all-cause survival was significantly associated with a *BRCA1* mutation (HR=4.73, 95%CI 1.45–15.43, *p*=0.01). This result is different than in our previously reported published analysis of 2270 ovarian cancer patients, in which we did not find an association of *BRCA1* mutation with overall survival [5]. However, it should be noted that low-grade ovarian cancers are rare and in that study, 7.3% of all cases (167 out of 2270) were accounted and only 4 cases carried *BRCA1* mutation.

Our study has several limitations. It is based on a relatively small number of cases, and obtained results should be considered as preliminary. In this study, we analyzed the 21 most common Polish founder mutations in 5 genes associated with breast/ovarian cancer risk. Therefore, we omitted other non-founder mutations which might be detected by full sequencing of these genes. In our study, the cases and controls were not matched. The time of recruitment and mean age of cases and control were similar. It is unlikely but it might be that other unknown genetic or lifestyle factors could cause a study bias.

## Conclusions

The results of our study suggest that *CHEK2* (c.470T>C) may possibly play a role in the pathogenesis of BOT, but not low-grade ovarian cancer. The presence of the *CHEK2* missense (c.470T>C) mutation may be associated with earlier age at diagnosis of BOT and about 10% worse rate of a 10-year survival. On contrary, the low-grade ovarian cancer is associated with *BRCA1* and *PALB2* mutations and presents worse survival among *BRCA1* carriers. These results may suggest that the genetic predisposition and the molecular mechanisms underlying tumor initiation differ between invasive and borderline tumors of the ovary*.*

Due to the low number of BOT patients, there remains the possibility that *CHEK2 *c.470T>C variant may not be directly related to tumor development, but rather may be a coincidental finding. Because of that, the obtained results should be considered as preliminary. Further studies conducted on larger groups are required to verify these observations.

## Data Availability

The data presented in this study are available from the corresponding author on reasonable request**.**

## Abbreviations

BOT: Borderline ovarian tumor
PTT: Protein truncating mutations

## References

[1] Hauptmann S, Friedrich K, Redline R, Avril S (2017). Ovarian borderline tumors in the 2014 WHO classification: evolving concepts and diagnostic criteria. Virchows Arch.

[2] Prat J (2017). Pathology of borderline and invasive cancers. Best Pract Res Clin Obstet Gynaecol.

[3] Fischerova D, Zikan M, Dundr P, Cibula D (2012). Diagnosis, treatment, and follow-up of borderline ovarian tumors. Oncologist.

[4] Sherman ME, Mink PJ, Curtis R, Cote TR, Brooks S, Hartge P, Devesa S (2004). Survival among women with borderline ovarian tumors and ovarian carcinoma: a population-based analysis. Cancer.

[5] Łukomska A, Menkiszak J, Gronwald J, Tomiczek-Szwiec J, Szwiec M, Jasiówka M, Blecharz P (2021). Recurrent mutations in BRCA1, BRCA2, RAD51C, PALB2 and CHEK2 in Polish patients with ovarian cancer. Cancers (Basel).

[6] Menkiszak J, Gronwald J, Górski B, Jakubowska A, Huzarski T, Byrski T, Foszczyńska-Kłoda M (2003). Hereditary ovarian cancer in Poland. Int J Cancer.

[7] Ramus SJ, Song H, Dicks E, Tyrer JP, Rosenthal AN, Intermaggio MP, Fraser L (2015). Germline mutations in the BRIP1, BARD1, PALB2, and NBN genes in women with ovarian cancer. J Natl Cancer Inst.

[8] Song H, Dicks EM, Tyrer J, Intermaggio M, Chenevix-Trench G, Bowtell DD, Traficante N (2021). Population-based targeted sequencing of 54 candidate genes identifies PALB2 as a susceptibility gene for high-grade serous ovarian cancer. J Med Genet.

[9] Song H, Dicks E, Ramus SJ, Tyrer JP, Intermaggio MP, Hayward J, Edlund CK (2015). Contribution of germline mutations in the RAD51B, RAD51C, and RAD51D genes to ovarian cancer in the population. J Clin Oncol.

[10] Gotlieb WH, Chetrit A, Menczer J, Hirsh-Yechezkel G, Lubin F, Friedman E, Modan B (2005). National Israel Ovarian Cancer Study Group. Demographic and genetic characteristics of patients with borderline ovarian tumors as compared to early stage invasive ovarian cancer. Gynecol Oncol.

[11] Gotlieb WH, Friedman E, Bar-Sade RB, Kruglikova A, Hirsh-Yechezkel G, Modan B, Inbar M (1998). Rates of Jewish ancestral mutations in BRCA1 and BRCA2 in borderline ovarian tumors. J Natl Cancer Inst.

[12] Bjørge T, Lie AK, Hovig E, Gislefoss RE, Hansen S, Jellum E, Langseth H (2004). BRCA1 mutations in ovarian cancer and borderline tumours in Norway: a nested case-control study. Br J Cancer.

[13] Risch HA, McLaughlin JR, Cole DE, Rosen B, Bradley L, Kwan E, Jack E (2001). Prevalence and penetrance of germline BRCA1 and BRCA2 mutations in a population series of 649 women with ovarian cancer. Am J Hum Genet.

[14] Lhotova K, Stolarova L, Zemankova P, Vocka M, Janatova M, Borecka M, Cerna M (2020). Multigene panel germline testing of 1333 Czech patients with ovarian cancer. Cancers (Basel).

[15] Szymanska-Pasternak J, Szymanska A, Medrek K, Imyanitov EN, Cybulski C, Gorski B, Magnowski P (2006). CHEK2 variants predispose to benign, borderline and low-grade invasive ovarian tumors. Gynecol Oncol.

[16] Park JM, Kim MK (2014). Hereditary risk evaluation for borderline ovarian tumors based on immunohistochemistry. J Menopausal Med.

[17] Stratton JF, Gayther SA, Russell P, Dearden J, Gore M, Blake P, Easton D (1997). Contribution of BRCA1 mutations to ovarian cancer. N Engl J Med.

[18] Szwiec M, Jakubowska A, Górski B, Huzarski T, Tomiczek-Szwiec J, Gronwald J, Dębniak T (2015). Recurrent mutations of BRCA1 and BRCA2 in Poland: an update. Clin Genet.

[19] Rogoża-Janiszewska E, Malińska K, Cybulski C, Jakubowska A, Gronwald J, Huzarski T, Lener M (2020). Prevalence of recurrent mutations predisposing to breast cancer in early-onset breast cancer patients from Poland. Cancers (Basel).

[20] Cybulski C, Kluźniak W, Huzarski T, Wokołorczyk D, Kashyap A, Rusak B, Stempa K (2019). The spectrum of mutations predisposing to familial breast cancer in Poland. Int J Cancer.

[21] Cybulski C, Górski B, Huzarski T, Masojć B, Mierzejewski M, Debniak T, Teodorczyk U (2004). CHEK2 is a multiorgan cancer susceptibility gene. Am J Hum Genet.

[22] Cybulski C, Kluźniak W, Huzarski T, Wokołorczyk D, Kashyap A, Jakubowska A, Szwiec M (2015). Clinical outcomes in women with breast cancer and a PALB2 mutation: a prospective cohort analysis. Lancet Oncol.

[23] Zagorianakou N, Stefanou D, Makrydimas G, Zagorianakou P, Briasoulis E, Karavasilis V, Pavlidis N (2006). Clinicopathological study of metallothionein immunohistochemical expression, in benign, borderline and malignant ovarian epithelial tumors. Histol Histopathol.

[24] Ozer H, Yenicesu G, Arici S, Cetin M, Tuncer E, Cetin A (2012). Immunohistochemistry with apoptotic-antiapoptotic proteins (p53, p21, bax, bcl-2), c-kit, telomerase, and metallothionein as a diagnostic aid in benign, borderline, and malignant serous and mucinous ovarian tumors. Diagn Pathol.

[25] Lahiri DK, Schnabel B (1993). DNA isolation by a rapid method from human blood samples: effects of MgCl2, EDTA, storage time, and temperature on DNA yield and quality. Biochem Genet.

[26] Tropé C, Kaern J, Davidson B (2012). Borderline ovarian tumors. Best practice & research. Clin Obstet Gynaecol.

[27] Bendifallah S, Ballester M, Uzan C, Fauvet R, Morice P, Darai E (2014). Nomogram to predict recurrence in patients with early- and advanced-stage mucinous and serous borderline ovarian tumors. Am J Obstet Gynecol.

[28] Kalapotharakos G, Högberg T, Bergfeldt K, Borgfeldt C (2016). Long-term survival in women with borderline ovarian tumors: a population-based survey of borderline ovarian tumors in Sweden 1960-2007. Acta Obstet Gynecol Scand.

[29] Bell DA (2005). Origins and molecular pathology of ovarian cancer. Mod Pathol.

[30] IeM S, Kurman RJ (2005). Molecular pathogenesis of ovarian borderline tumors: new insights and old challenges. Clin Cancer Res.

[31] Cybulski C, Huzarski T, Górski B, Masojć B, Mierzejewski M, Debniak T, Gliniewicz B (2004). A novel founder CHEK2 mutation is associated with increased prostate cancer risk. Cancer Res.

[32] Myszka A, Karpinski P, Slezak R, Czemarmazowicz H, Stembalska A, Gil J, Laczmanska I (2011). Irrelevance of CHEK2 variants to diagnosis of breast/ovarian cancer predisposition in Polish cohort. J Appl Genet.

[33] Verbruggen MB, Zweemer RP, Piek JM, van Unnik GA, van Diest PJ, Gille JJ, Menko FH (2007). A case of loss of heterozygosity in the BRCA2 gene of a borderline ovarian tumor: case report and review of literature. Int J Gynecol Cancer.

[34] Chen J, Chang C, Huang HC, Chung YC, Huang HJ, Liou WS, Chiang AJ (2015). Differentiating between borderline and invasive malignancies in ovarian tumors using a multivariate logistic regression model. Taiwan J Obstet Gynecol.

[35] Grabowska-Derlatka L, Derlatka P, Palczewski P, Danska-Bidzinska A, Pacho R (2013). Differentiation of ovarian cancers from borderline ovarian tumors on the basis of evaluation of tumor vascularity in multi-row detector computed tomography--comparison with histopathology. Int J Gynecol Cancer.

[36] Skírnisdóttir I, Garmo H, Wilander E, Holmberg L (2008). Borderline ovarian tumors in Sweden 1960-2005: trends in incidence and age at diagnosis compared to ovarian cancer. Int J Cancer.

[37] Morice P (2006). Borderline tumours of the ovary and fertility. Eur J Cancer.

[38] Tropé C, Davidson B, Paulsen T, Abeler VM, Kaern J (2009). Diagnosis and treatment of borderline ovarian neoplasms ‶the state of the art″. Eur J Gynaecol Oncol.

[39] Trimble CL, Kosary C, Trimble EL (2002). Long-term survival and patterns of care in women with ovarian tumors of low malignant potential. Gynecol Oncol.

[40] Baldwin LA, Huang B, Miller RW, Tucker T, Goodrich ST, Podzielinski I, DeSimone CP (2012). Ten-year relative survival for epithelial ovarian cancer. Obstet Gynecol.

